# IFND: a benchmark dataset for fake news detection

**DOI:** 10.1007/s40747-021-00552-1

**Published:** 2021-10-16

**Authors:** Dilip Kumar Sharma, Sonal Garg

**Affiliations:** GLA University, Mathura, India

**Keywords:** Deep-learning, Fake news detection, Indian dataset, LDA topic modelling, Machine learning

## Abstract

Spotting fake news is a critical problem nowadays. Social media are responsible for propagating fake news. Fake news propagated over digital platforms generates confusion as well as induce biased perspectives in people. Detection of misinformation over the digital platform is essential to mitigate its adverse impact. Many approaches have been implemented in recent years. Despite the productive work, fake news identification poses many challenges due to the lack of a comprehensive publicly available benchmark dataset. There is no large-scale dataset that consists of Indian news only. So, this paper presents IFND (Indian fake news dataset) dataset. The dataset consists of both text and images. The majority of the content in the dataset is about events from the year 2013 to the year 2021. Dataset content is scrapped using the Parsehub tool. To increase the size of the fake news in the dataset, an intelligent augmentation algorithm is used. An intelligent augmentation algorithm generates meaningful fake news statements. The latent Dirichlet allocation (LDA) technique is employed for topic modelling to assign the categories to news statements. Various machine learning and deep-learning classifiers are implemented on text and image modality to observe the proposed IFND dataset's performance. A multi-modal approach is also proposed, which considers both textual and visual features for fake news detection. The proposed IFND dataset achieved satisfactory results. This study affirms that the accessibility of such a huge dataset can actuate research in this laborious exploration issue and lead to better prediction models.

## Introduction

Fake news can proliferate exponentially in the early stages on a digital platform which can cause major adverse societal effects. Therefore, it is required to detect fake news as early as possible. Fake news can affect the mental health of children and adults along with physical health. Artificial intelligence [1] could help doctors in making decisions based on patient's behavioural data and the use of social media. Authors in paper [2] discussed various wearable health-monitoring devices to monitor the human body. They used Internet of things techniques which will help in daily health management. An increase in the Internet of things' use also increases the concept of smart technologies, such as self-driving and self-monitoring [3]. The implementation of the Internet of things is difficult because of the need for fog and edge computing [4]. It is essential to check the authenticity of news to nab misinformation dissemination. Users generally believe in the appealing headline and the image because of time constraints. Thus, sensational headlines generate misunderstood, falsified pieces of information. Fake news detection is an arduous task. There are various reasons to create fake news, like the deception of personalities and creating biased views to change the outcome of important political events. Man-kind struggles with unprecedented fear and dependency on social media in this COVID-19 situation, resulting in the surge of fake news [5].

**Fig. 1:**
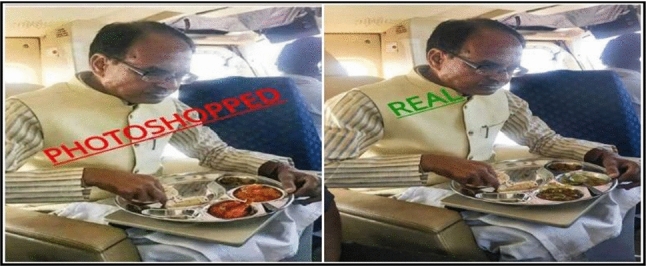
Morphed image shared to claim Former MP CM Shivraj Chouhan was eating non-veg [10]

India is a developing country. We are being been bombarded with rumours. People are unaware of what is accurate, and now it is a matter of life and death. There is a need to develop an automatic algorithm to detect fake content in the healthcare domain. Fake news also affects the physical health of citizens and medical professionals. False information creates lynching of innocents, which emerged as a new trend in India. Social media accelerated gossip and hearsay to the public. An MIT study reveals that fake content propagates six times faster than the original content on Twitter [6]. This is because false content helps in generating more money than the truth. For example, let us consider the Myammar case [7]; these online platforms help initiate violence and manipulate public opinions regarding a particular event. Fake news creates chaos amongst people. Fake information resulted in mass killings. Other hazards include a large number of rapes and the burning of residence of people. This violence forced the 700,000 Rohingya Muslims having to flee the country. It is not that highly motivated propagandists also exist before, but nowadays, these digital platforms are responsible for fast propagating fake news without much money. There are various reasons for fake news generation like hostile intention, fabricating political profits, defacing the business, cause conflicts, personal agenda, entertainment, frenzy or commitment, the influence of governing authority, etc. So it is required to take action against the one who spreads fake news. Various prominent companies Adobe, Facebook, and Google, are involved in developing tools to control the propagation of fake content over social media. Various tools and extensions are suggested by authors in the paper [8] to control fake news propagation. Authors in paper [9] used the primary reproduction number to analyze the messages propagated on social media. They also suggested the control mechanism for this dubious message dissemination. Figure 1 represents the morphed image of Shivraj Chouhan, which became viral on social media with the false claim that Shivraj Chouhan was eating non-veg food.

This paper presents an Indian news dataset from the year 2013 to the year 2021. Various news websites are scrapped using the Parsehub tool to gather reliable data. Fake data are limited, so; this paper introduced an intelligent text augmentation algorithm to increase the size of fake articles.

In a nutshell, the major contributions of this paper are described below:

- This paper introduces a benchmark Indian news dataset for fake news identification. This is the first large-scale publicly available dataset in the Indian context.
- This paper introduces a novel approach to text augmentation. Fake text is augmented by extracting a similar bag of words by applying the cosine similarity approach, which helps machine learning and deep-learning models to produce more accurate results.
- The model employs VGG16 and Resnet-50 model for images analysis and applied several machines and deep-learning models for text analysis.
- A multi-modal approach using LSTM for text analysis and VGG16 for image analysis is used.
- To determine the category of news, latent Dirichlet allocation (LDA) topic modelling is applied.
- Study and analyze the trends of fake news using the latest dataset created using fact-checking websites from the year 2013–2021 from events of India.

This paper has the following sections. “Related work” discusses related work. “Proposed method” elaborates the proposed methodology used for dataset creation. “Analysis of the dataset” provides existing prominent datasets and analysis of a proposed dataset. “Results analysis” presents experimental results and comparative analysis with other models. “Applications” shows the area where the proposed dataset can be applied, and “Conclusion” discusses the conclusion and future work possible.

## Related work

In recent years, many techniques for fake news detection had been proposed. There are various challenges like the existence of echo chamber, limitation of benchmark dataset, and deceptive writing style that makes this task more cumbersome.

### Text features

The text of the news article is the most important part. Many existing methods used textual features for fake news identification. Statistical or semantic features are also extracted for fake news detection.

Authors in the study [11] used informativeness, readability, and subjectivity characteristics for shill review detection. For real review, data are collected from amazon.com. Shill reviews were related to the MP3 player domain. Authors in paper [12] employ sentiment words as a text feature. They found that more sentiment words in tweets generally indicate more non-credible information. Latent Dirichlet allocation (LDA) [13] topic modelling is used by authors in the paper [14] to determine the topic of online posts. They characterize the Websites and reputations of the publishers of the hundreds of news articles. They also explored the essential terms of each news and their word embeddings. Word embedding proves to be useful in fake news detection. Authors in the paper [15] presented 234 stylometric features considering linguistic and syntactic features for false review detection. They had used a machine learning classifier named support vector machine (SVM) with sequential minimal optimization (SMO) and Naive Bayes. They used an opinion spam corpus with gold-standard deceptive opinions. The corpus consists of 1600 reviews. The *F*-measure value of the SMO classifier is 84%, and Naive Bayes is 74%. The best result for both classifiers is obtained by applying SMO classifier on lexical and syntactic combinations. There is a need to extract content-specific features and find the effects of combining the content-specific features with syntactic and lexical features to improve the accuracy.

*n*-gram feature extraction technique with machine learning approach is also applied by authors in paper [16] for fake news identification. They concluded that SVM achieved the best result in the machine learning classifier by achieving 92% accuracy. Moreover, a larger value of *n*-gram could create an adverse impact on accuracy [17]. Authors in paper [18] applied 57 linguistic features. They used word2vec embedding on a dataset of size almost 4000 articles. All features are used in a single linguistic feature set. The proposed algorithm achieved 95% accuracy. To improve the result obtained by authors in [18], a new model named WELFAKE is suggested by authors in [19]. They used 20 linguistic features instead of 57 and then combined these features with word embeddings and implemented voting classification. This model is based on count vectorizer and Tf-idf word embedding and used a machine learning classifier. For unbiased dataset creation, they merged four existing datasets named Kaggle, McIntire, Reuters, and BuzzFeed; the WELFake model achieved 96.73% accuracy on the WELFake dataset. SVM classifier produced the best result in comparison to other ML models.

Authors in paper [3] used text, user-specific, and message-specific features for hoax detection using the Italian Facebook dataset. They have applied various machine learning models, including logistic regression and linear regression. The proposed approach achieved 91% accuracy using linear regression. Another work by authors in paper [20] uses the same features text, user, and message to determine the credibility of 489 330 Twitter accounts. They used Random Forest, Decision Tree, Naive Bayes, and feature-rank NB algorithms with a tenfold cross-validation strategy. A new dataset named Newpolitifact is proposed by authors in the study [21] for fake news identification. This dataset is created by scraping the Politifact.com website. The size of this dataset is small. The authors applied various machine learning classifiers to analyze the performance of the dataset. The limitation of the machine learning model lies in need for manual feature engineering and an extensive training dataset. ML classifiers work best for the ML settings they were initially designed for [22]. So, no single classifier guaranteed the best results for all datasets. To cover complex features of models, researchers’ gas started using deep-learning techniques. Authors in paper [23] suggested a deep convolutional neural network named the fake news detection network (FNDNet) model. They used the Kaggle data set. The proposed approach obtained 98.36% accuracy. But, the limitation of the proposed approach is that they did not consider generalized text [18].

Authors in paper [24] proposed a chrome extension-based approach for fake news identification on the Facebook platform. They applied both machine learning and deep-learning classifiers. Using LSTM, the proposed approach achieved the highest of 99.4% accuracy in comparison to machine learning classifiers. The approach helps in determining fake news in real time on user's chrome environment by examining user profiles and shared posts. Authors in paper [25] introduced an approach called TraceMiner to identify fake news. TraceMiner takes a trace of the message and then classifies the category. They used the LSTM-RNN model for classification. TraceMiner utilized the network structure like proximity of node and social dimension information. In further research, it is possible to use the TraceMiner for other network mining tasks like a recommendation and link prediction.

Authors in paper [26] worked on the generalization of the model. They used a hybrid of convolutional and recurrent neural networks. FA-KES and ISOT datasets are used for experiments. They achieved 50% generalization accuracy. There is a need to improve the structure of the model to improve cross-validation generalizations. Authors in paper [27] integrated CNN and Bi-LSTM model with attention mechanism for fake news identification. Glove word embedding is applied to generate vector representation. They used the LIAR dataset for implementation. The proposed hybrid approach obtained 35.1% accuracy. These discussed models are good but based only on text. There is a limitation where fake images can be left from detection. The news headings may be real or relevant, but the images are posted to deceive people.

#### Text dataset

There are various text datasets that researchers used for fake news detection. Some are described below:

- *BuzzFeedNews* This small dataset is developed using Facebook. Five Buzzfeed journalist’s fact-check the required dataset. It only contains the headlines and texts of 2282 posts [28]
- *BuzzFace* This dataset consists of four categories named mostly true, mostly false, a mixture of true and false, and no factual information. The dataset contains various features, such as body text, images, links, and Facebook plugin comments. It is formed of total of 2263 articles [29]
- *LIAR* This dataset [30] is collaborated using the API of a fact-checking website named Politifact. This dataset contains a variety of fine-grained articles into six categories: pants-fire, false, barely true, half-true, mostly true, and true. It is formed of 12,836 short statements rather than the complete text.
- *CREDBANK* This dataset is formed for explicitly targeting the Twitter news feed. Moreover, it contains crowdsourced data from 60 million tweets. Data is distributed in four files. Thirty annotators did the verification of 1000 topics [31]. Tweets are categorized into 1049 incidents with a 30-dimension vector of truthfulness classes, using a 5-point Likert scale.
- *Reuters* [16] This dataset consists of 7769 training documents, and for testing, the size is 3019. All these documents are collected from a single source. It is a multi-label dataset and provides 90 classes. News is collected from a single source which can increase the chances of biased data.
- *McIntire* [32] This is a binary labelled dataset with True and Fake label. It consists of 10,558 rows and 4 columns. It consists of real news from both left and right wing.
- *FacebookHoax* [33] This dataset contains 15,500 Facebook posts. The number of unique users is 909,236.
- *Kaggle* [34] contains true news and fake news data, but source information is missing.

### Image and multi-modal features

The noisy content on social media makes the fake news identification task difficult. At present, researchers have initiated the use of image features along with text for phony news detection. Authors in paper [35] used a convolutional neural network for fake image detection. Gradient weighted class activation mapping is applied for heatmap creation. This proposed model achieved 92.3% accuracy on the CASIA dataset. Authors in paper [36] investigated a self-trained semi-supervised deep-learning algorithm to increase the performance of neural networks. They have used the confidence network layer, but the proposed model achieved less accuracy when the input image is from social media.

Yang et al. [37] developed a model TI-CNN (Text and Image information based Convolutional Neural Network) integrating text and images. They used two parallel CNNs to extract hidden features from both text and images. They worked on a dataset collected from online websites. The dataset covers almost 20,000 news. The proposed approach obtained a 0.921 *F*-score. Authors in paper [24] analyzed multiple features of Facebook account for fake news detection. They used a deep-learning classifier (DL) to measure the performance. Gupta et al. [38] proposed MVAE (Multimodal Variational Autoencoder) model. It uses RNN and Bi-LSTM for text analysis. VGG19 model is adopted for image classification. The Authors used Twitter [39] and Weibo datasets for experiments. They achieved 74.5% accuracy using the Twitter dataset and 82.4% accuracy using the Weibo dataset. Another framework called SAFE (Similarity Aware Fake news detection method) is introduced in the paper [40]. This method used a multi-modal feature that includes textual and visual features for the detection of false information. The neural network is adopted to extract multi-modal features independently; then,—the relationship is predicted. Authors concluded that while writing fake news, writers use attractive but irrelevant images, and it is difficult to identify real and manipulated images to match the fake text. Another work using a multi-modal approach is introduced in the paper [41]. Researchers applied sentence transformers for text analysis and applied EfficientNetB3 for image analysis. These two different layers are fused to obtain the final accuracy. The authors also used the ELA technique to find the manipulated part of the image. This model achieved an accuracy of 79.50 on the Twitter dataset and 80% on the Weibo dataset. Another prominent model is presented in the paper [42] named Spotfake for fake news identification. They used multiple channels to learn intrinsic features. The authors used VGG19 for image analysis and Bert architecture to learn text features. They combined these features to obtain the final prediction. This model performed better than the above-discussed state of art model by obtaining an accuracy of 77% on the Twitter dataset and 89.2% on the Weibo dataset.

The trade-off using the multi-modal approach is time complexity. A large amount of time is required during training and fitting the model because it combines two different modules for binary prediction. Learning correlation in text and image and event discriminator is also another limitation while using a multi-modal approach.

Therefore, in the proposed work, the problem mentioned above is solved using the following:

- Both machine-learning and deep-learning classifiers are used for text classification.
- Augmentation techniques increase the accuracy of the proposed model.
- Using VGG16 in multi-modal classification as VGG16 replaces a large number of hyper-parameters with a convolutional layer of 3*3 filters with stride 1. They have proven to provide higher accuracy with limited parameters for the image classification task.
- The model is independent of any sub-activities for prediction.

#### Image and multi-modal Dataset

There are various datasets that consist of images and text. Some are described below:

- *CASIA* This dataset is generally applicable for image tampering detection. It exists in CASIAv1and CASIAv2. CASIA v1 contains 921 images, while version two has 5123 images [43].
- *Weibo* This dataset consists of 4, 664 events, 2.8 million users, and 3.8 million posts. The binary label is used. This dataset covers event from the year 2012 to 2016. Both web and mobile platforms are used to create the dataset. Has domestic and international new [44].
- *Medieval* This dataset consists of a total of 413 images. It consists of 193 real images and 218 fake images. Two manipulated videos also exist. Images are associated with 9404 fake tweets posted by 90,025 unique users and 6225 real tweets by 5895 unique users [45].
- *FakeNewsNet* This dataset is collected from two fact-checking websites named Politifact and GossipCop. From Politifact, 447 true news and 336 fake news are collected, and 16,767 true, and 1650 fake news are extracted from Gossipcop [46].

### Resource scarce language existing dataset

In recent work, authors in paper [47], introduced two new datasets. The first one was prepared by manually scrapping real and fake news from various websites, and the second was prepared using a data augmentation algorithm. Another dataset is proposed in paper [19] named WELFake dataset by incorporating existing four datasets with approximately 72,000 articles. There are datasets introduced for resource-scarce language. In paper [48], the authors proposed a dataset of approx. 50 K news. This dataset contains all the clickbait, satire news, and misleading news. They proposed a system to identify Bangla's fake news. They used traditional linguistic features and neural models for implementation. Results depicted that linear classifiers with linguistic approaches worked better in comparison to the neural model. In paper [49], researchers discussed the significance of readability features for fake content identification. They used the Brazilian Portuguese language. Readability features usually measure the number of complex words, long words, syllables, grade level, and text cohesion. It generally considers all linguistic levels for determining readability features. This feature achieved 92% accuracy. In the paper [50], the authors try to find the impact of the machine translation to text data augmentation for the fake new identification in Urdu. Urdu is a resource-scarce language. This text augmentation helps in training improvement when less dataset is available. Results demonstrated that the classifier trained on the original Urdu dataset performed better than the purely MT-translated and the augmented (the combination of the two) datasets despite the 20% size increase in the augmented dataset. Authors in paper [51] suggested a two-level attention-based deep neural network model for phony news detection. To conduct experiments, they used a corpus of Bulgarian news. Authors in paper [52] presented a 174 truthful and deceptive News articles dataset in Russian. In a study [53], authors developed the first multi-lingual cross-domain dataset. Dataset covered of 40 languages of 105 countries. The total size of the dataset was 5182 fact-checked news articles. Authors in paper [54] presented a benchmark Spanish fake news dataset. They created the dataset for health news. They proposed a novel framework consists of structure layer and veracity layer. Generally, fake news research is limited to specific social networks and languages. So, these works highlighted the research and helped us get a more in-depth understanding of fake news and the need to create an IFND dataset. There are three reasons for creating the IFND dataset—(1) the limitation of labelled data [30], (2) the writing style differs from region to region [55] so, a specific Indian context dataset is required and (3) above-mentioned previous datasets do not include news from multiple news sources, IFND resolves this issue and consists of multi-modal information from multiple news sources.

## Proposed method

There is no Indian dataset available, so we proposed an IFND dataset specific to Indian news to bridge this research gap. We scraped real news from various trusted websites, such as Times Now news [56] and The Indian Express [57], to build our dataset. We collect fake news from multiple websites like Alt news [58], Boom live [59], digit eye [60], The logical Indian [61], News mobile [62], India Today [63], News meter [64], Factcrescendo [65] and Afp [66].

**Fig. 2:**
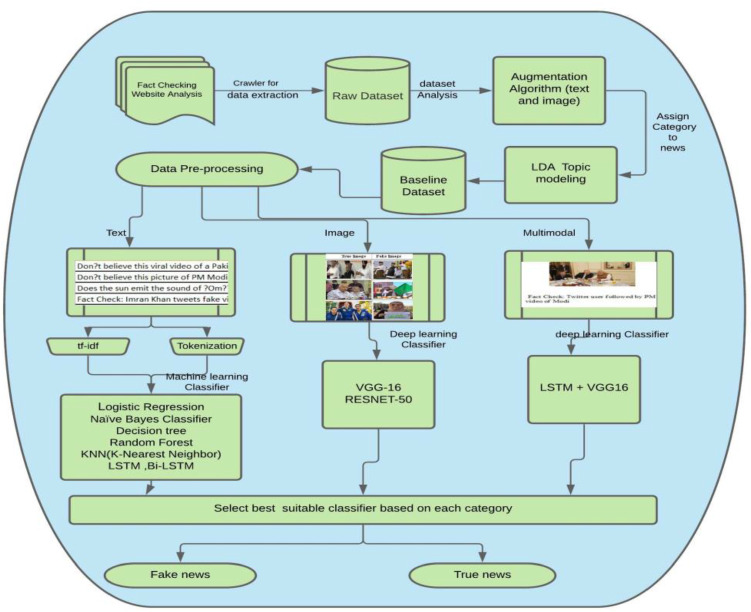
Proposed framework for IFND dataset


To create a dataset, we have used the Parsehub scrapper, a tool that is used from scrapping a website. The Indian dataset comprises 56,868 news. The true news is collected from Tribune [67], Times Now news, The Statesman [68], NDTV [69], DNA India [70], and The Indian express. The fake news has been scraped from Alt news, Boomlive, digit eye, The logical Indian, News mobile, India Today, News meter, Factcrescendo, TeekhiMirchi [71], Daapan [72], and Afp publishes articles on international, national, and local news. We have preferred to collect the news from a fact-checked column of news websites, such as Alt news and Boomlive, and check the label of each news manually before putting the news in a particular category. We have gathered the news from the year 2013 to the year 2021. To fetch news related to India, we select only the India news column, and we have also created a filter to remove other news. We have manually asked several subject annotators to cross-verify the dataset that we collect. We have collected the information of various fields like Title of news, Date and Time, Source of news, Link of news, Image link, and Label (True/Fake). The category of news is also included using LDA topic modelling. There are five categories—Election, Politics, COVID-19, Violence and Miscellaneous-derived using LDA topic modelling. Figure 2 shows the proposed working framework used for the creation of the IFND dataset. First, various fact-checking websites are scrapped to collect the news; then, fake news is limited. So to create a biased dataset, a data augmentation technique is used. After data augmentation, the news is categorized into different categories using LDA topic modelling. Pre-processing is applied to the resulting dataset. Then different machine learning and deep-learning classifiers are used for both text and image analysis. A multi-modal approach is also applied by combining textual and visual features for fake news detection.

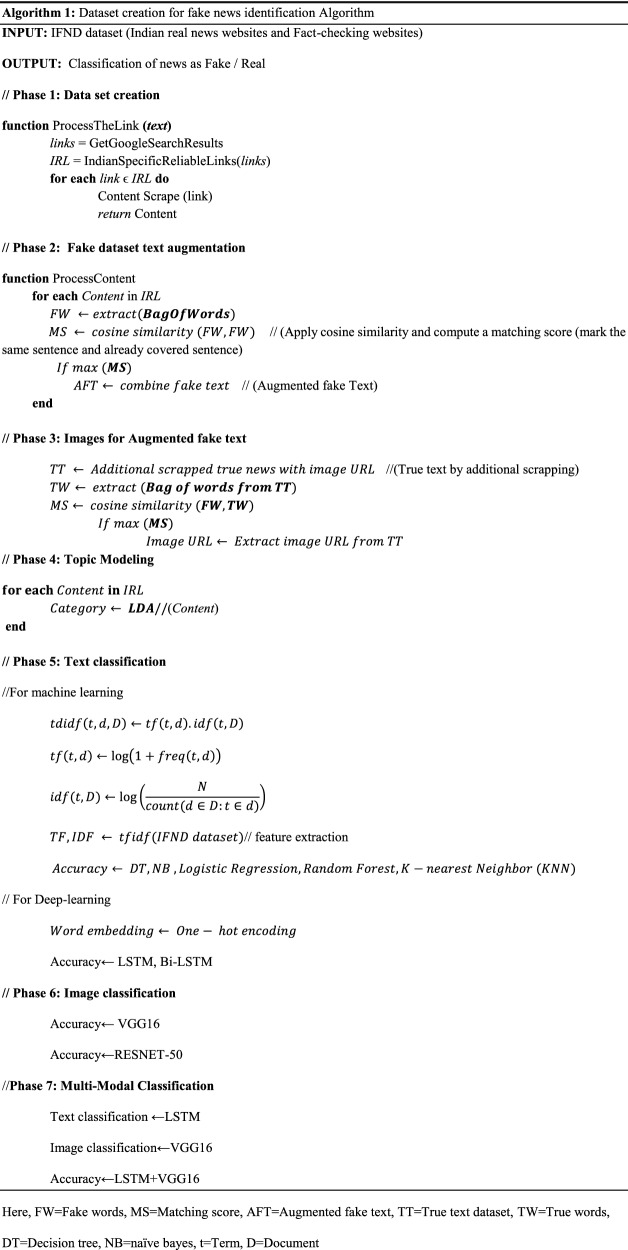


**Fig. 3:**
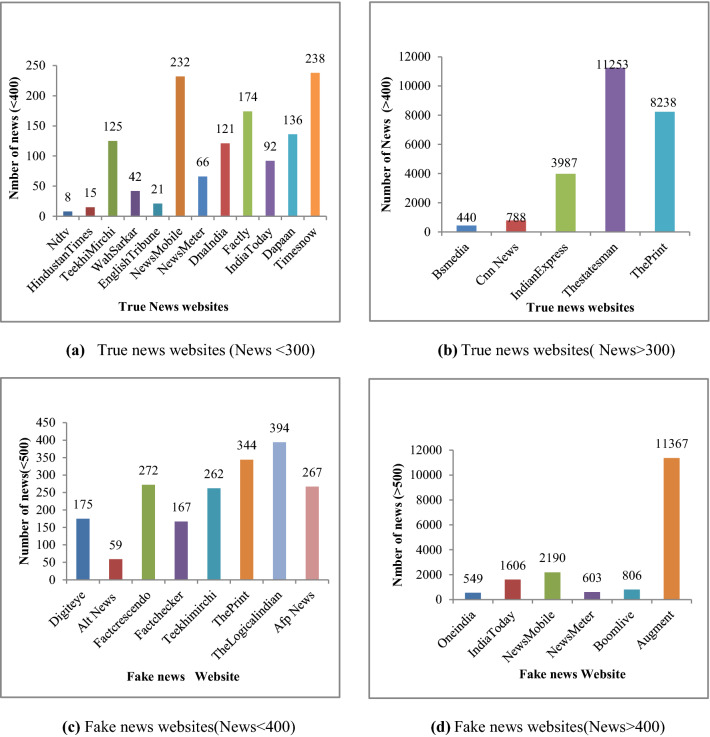
Statistics of IFND dataset

Figure 3a represents the real news website statistics of scrapped content less than 400. Figure 3b represents the list of websites from where more than 400 news is scrapped. Figure 3c shows the statistics of websites responsible for scrapping less than 500 fake news. Figure 3d represents the fact-checking websites for fake news contributing more than 500 data.

Table 1 shows the attributes of our proposed dataset. This dataset consists of id, news headings, image link, source, category, date, and label columns. This dataset supports binary classification. The label must be either true or fake. Moreover, Table 2 illustrates the images information of our IFND dataset. All these images are resized to 256*256 dimensions.

### Augmentation algorithm

**Table 1:** Snapshot of the proposed dataset

ID	Statement	Image	Web	Category	Date	Label
1	Fact Check: 1938 video of BKS Iyengar shared as PM Modi performing yoga	https://akm-img-a-in.tosshub.com/indiatoday/images/story/202011/Screenshot_20201124-232700_0-170x96.jpeg?yPyj5w40idCAv3WfDfdIiPVQ8jA67En9	INDIA TODAY	COVID-19	Nov 2020	Fake
2	Bihar Assembly Election 2020: This is why Tej Pratap shifted from Mahua to Hasanpur	https://cdn.dnaindia.com/sites/default/files/styles/third/public/2020/10/13/931041-tej-pratap-yadav-rabri-devi.jpg	DNAINDIA	ELECTION	Oct-20	True
3	Hathras case: CBI reaches victim's village, visits the crime scene	https://cdn.dnaindia.com/sites/default/files/styles/third/public/2020/10/13/931043-hathras-cbi.jpg	DNAINDIA	VIOLENCE	Oct-20	True

**Table 2:** Screenshot of proposed dataset image

	True image	Fake image
Image 1		
Image 2		
Image 3		

**Fig. 4:**
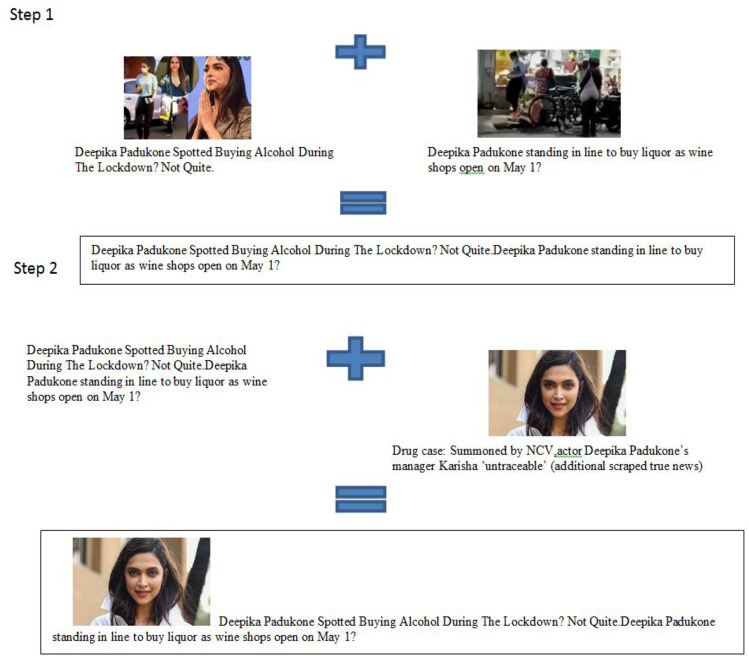
Example of intelligent augmentation technique

After scrapping, real news size is 37,809, while the size of fake news is 7271. So, to increase the size of the fake news dataset, there is a need for augmentation techniques. Figure 4 represents the example of the proposed augmentation algorithm. There are various ways to generate more content, like using the LSTM technique, more sentences can be generated, but the generated sentences are not much meaningful. So we used the following algorithm for fake text generation:

- All common bag of words is extracted from fake news, and then cosine similarity is calculated. The sentences are combined based on a matching score. This combined sentence is treated as a new fake statement, and all these sentences are marked so that we cannot use that sentences further.
- To retrieve the image of this augmented dataset, we performed text matching (cosine similarity) using a real news dataset. We have scrapped an additional 20,000 real news datasets, so the proposed dataset is not biased.

## Analysis of the dataset

**Fig. 5:**
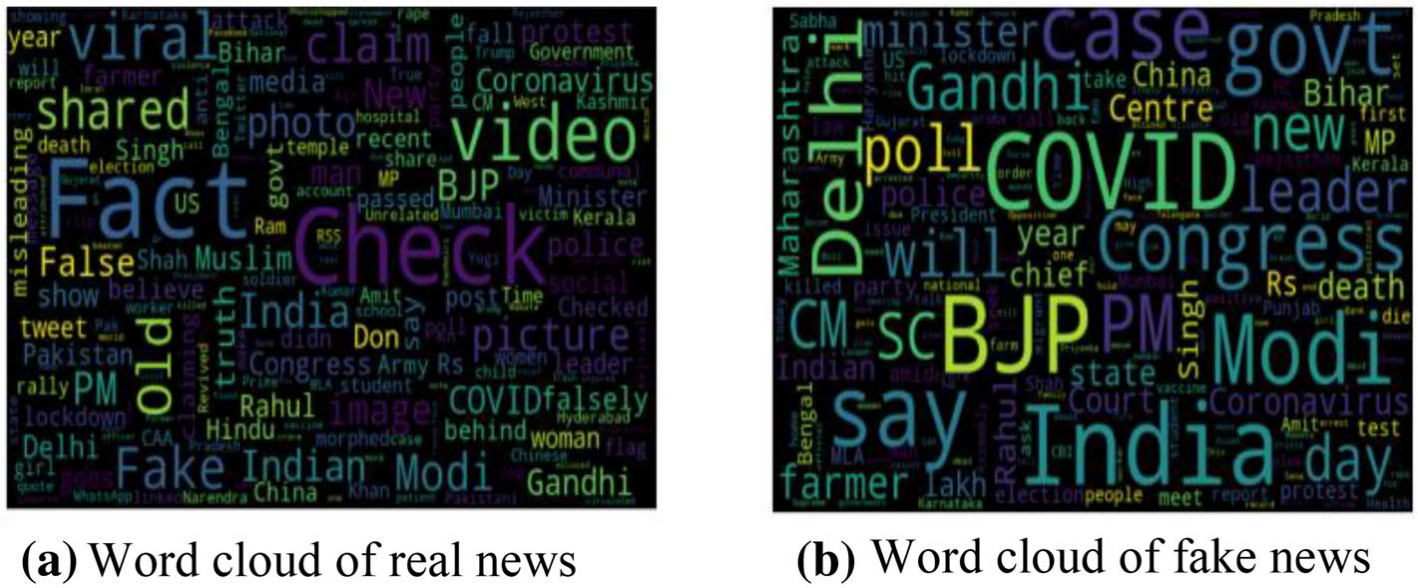
**a** Word cloud of real news. **b** Word cloud of fake news

**Fig. 6:**
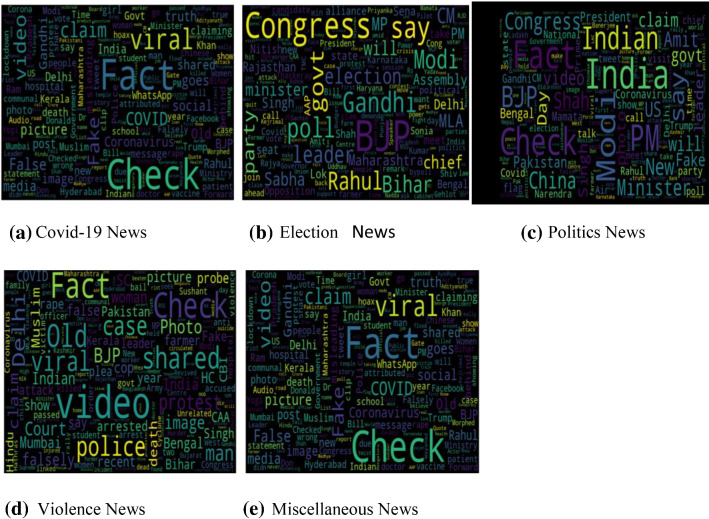
Word cloud of COVID-19 news (**a**). **b** Word cloud of election category. **c** Word cloud of politics category. **d** Word cloud of violence category. **e** Word cloud of miscellaneous category

One of the main reasons for creating our dataset is the absence of any large-scale Indian dataset. Big data can play an important role in academia to make evidence-based decisions [73]. To visualize the news content of the dataset, word cloud representations are used. Word cloud representations depict the frequency of the terms in a specific dataset. We draw some exciting conclusions from the word cloud representation shown in Fig. 5a, b. Real news word cloud represents important entities that occurred in actual events like the farmer, COVID, and Gandhi, while fake news word cloud highlights fake entities, such as old pictures, shared, morphed pictures. It was interesting to observe that fake news generally uses appealing headlines and does not have specific content that denotes real news. It is observed that both fake and real news articles are generally related to a political campaign. The length of statements in fake and real news is also dependent on the source of news from where they are scraped, irrespective of its behaviour. Images are also an essential factor in differentiating between real news and fake news. So, different classifiers are applied to the image also. Table 3 shows the comparison with existing datasets. It can be clearly seen that IFND has the most comprehensive news collection of both text and images. This dataset also has the novelty of being created from multiple news data sources. Also, this dataset is publicly available for all research fraternity. Figure 6 represents the word cloud of five categories obtained after applying LDA topic modelling.

**Table 3:** Comparison of existing datasets

Dataset	Total true news	Total fake news	Images used	Public availability
BuzzFeedNews [28]	826	901	No	Yes
BuzzFace [29]	1656	607	No	Yes
Weibo [44]	4779	4749	Yes	Yes
Twitter [39]	6026	7898	Yes	Yes
LIAR [74]	6400	6400	No	Yes
FacebookHoax [33]	6577	8923	No	Yes
FakeNewsNet [46]	18,000	6,000	Yes	Yes
Proposed-dataset	37,809	19,059	Yes	Yes

**Fig. 7:**
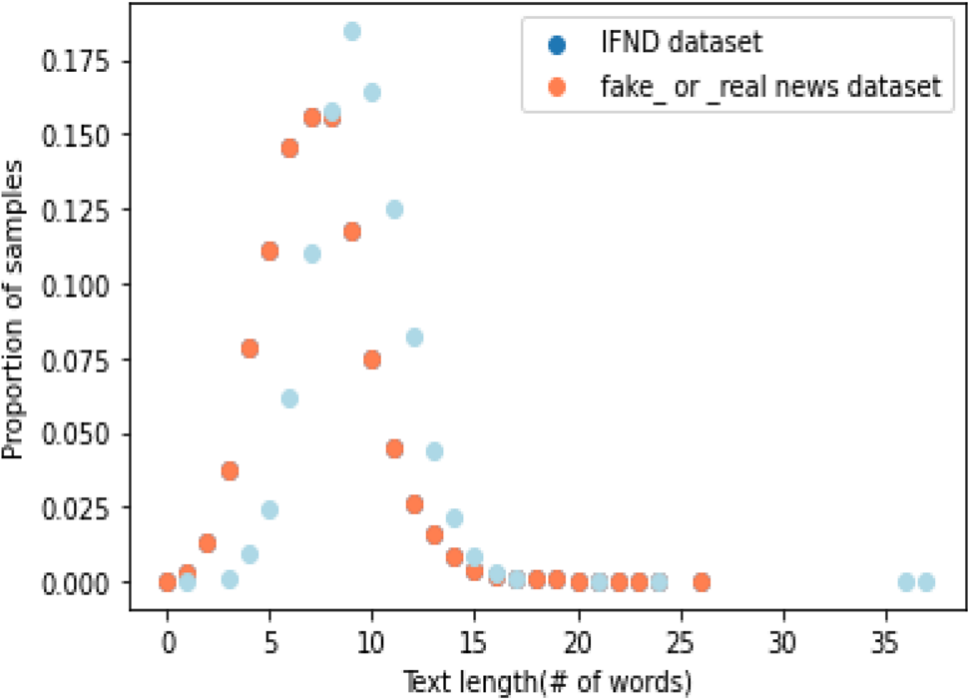
Comparison of word length distribution

Figure 7 represents the comparison of our proposed IFND dataset with George McIntire's fake_or_real news dataset [75]. To compare word length distribution, we took the length of the dataset similar to the fake_or_real news dataset. The title column of the fake_or_real news dataset is compared with the statement column of the IFND dataset. This graph clearly depicts that IFND dataset consists of longer sentence headlines in comparison to the existing dataset.

**Fig. 8:**
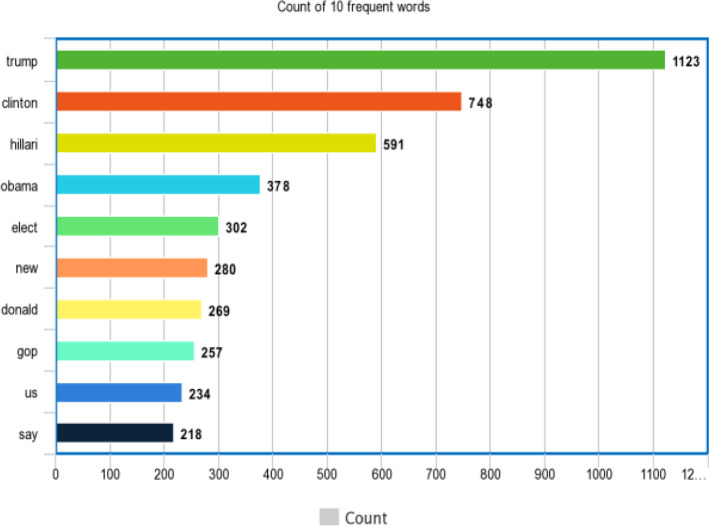
Most common 10 words of fake_or _real news dataset

**Fig. 9:**
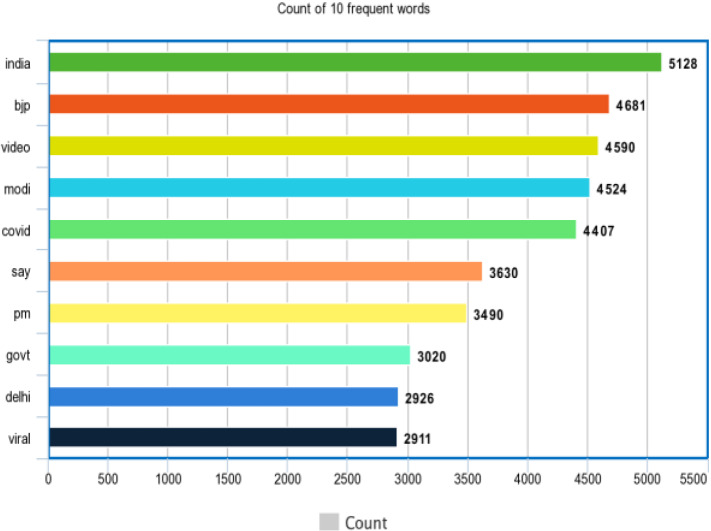
Most common 10 words of the IFND dataset

Figures 8 and 9 represent the top 10 most occurring words of the dataset.

### Text classification

For text feature extraction, first, we applied to pre-process techniques like removing stop words. Stemming was performed using snowball stemmer [76] to convert words into root form. Number values are removed using the IsNull() function of python. Punctuations, special symbols are also removed. Dataset is converted to lower case for further processing. The duplicate statement is released by applying the identical remove function.

#### Machine learning model

This section presents various machine learning models used in our experiments.

- *Naive Bayes* It is generally used for classification problems. It works well when the training dataset size is large. In various real-world applications like distinguishing spam mail from ham mail, an SVM classifier could be used. It assumes that the occurrence of a certain feature is not related to the occurrence of other features.
- *Decision Tree* This algorithm can be used for prediction and classification. Its work is based on rules.
- *Random Forest* It combines various decision trees and computes average results. The accuracy is dependent on the number of trees is used in the algorithm.
- *Logistic regression* It uses a logistic function for binary classification.
- *K-nearest neighbor* It is entirely dependent on the number of cases and available data as a neighbour. This algorithm is a lazy learning method that's by this one is very effective to classify the data and regression. The output is based on the number of a majority vote for classification using the mean, mode method among the K-nearest neighbours in the space.

For feature extraction, the term frequency-inverse document frequency (Tf-idf) algorithm is used. This algorithm is used by search algorithm to compute the document relevance based on scoring. Tf-idf is used to predict the significance of a term in a given document. It is calculated using:

1 $${tf}_{i,j}=\frac{{n}_{i,j}}{\sum_{k}{n}_{i,j}},$$

where *tf*_*i,j*_ is the number of occurrences of *i* in *j*, *tf*(*w*) = (frequency of word *w* appears in a document/total count words in the document).

2 $$ idf\left( t \right) = \log \frac{N}{{df_{t} }}, $$

where *N* is the total number of documents and *df*_*t*_ is the number of documents with the term *t*.

#### Deep-learning classifier

**Fig. 10:**
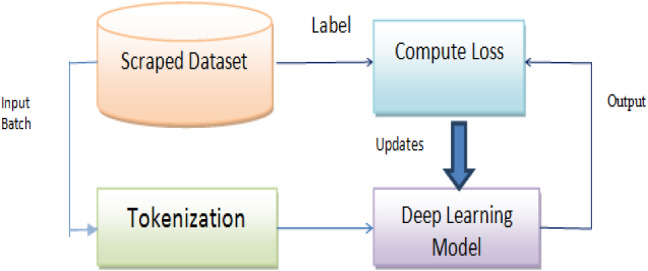
The architecture of deep-learning classifier

Deep-learning models are generally used in artificial intelligence applications. This section provides the detail of the LSTM and Bi-LSTM model to compute the results. Pre-trained word embedding is applied to make the sentence length equal. Figure 10 represents the general working architecture of the deep-learning model. In the deep-learning model, the first dataset is uploaded. Pre-processing is applied to remove unnecessary details. Tokenization is performed on the preprocessed dataset. We divide our dataset into a 67:33 ratio. 67% is used for training, and 33% is for testing purposes. After this, various deep-learning models are implemented and based on the model's outcome, the loss is predicted. Tables 4, 5 and 6 represent the layered architecture of the LSTM and Bi-LSTM model.

**Table 4:** LSTM layered architecture

Layer (type)	Output shape	Parameter number
Embedding	(None, 50, 40)	200,000
Dropout	(None, 50, 40)	0
LSTM	(None, 100)	56,400
Dense	(None, 1)	101
Total parameters: 256,501		
Trainable parameters: 256,501		
Non-trainable parameters: 0

**Table 5:** Hyper-parameter settings for LSTM

Hyper-parameter	Values
Number of dense layers	1
Dropout rate	0.3
Optimizer	Adam
Activation function	Sigmoid
Loss function	Binary cross-entropy
Number of epochs	10
Batch size	64

**Table 6:** Bi-LSTM layered architecture

Layer (type)	Output shape	Parameter number
Embedding	(None, 50, 40)	200,000
Dropout	(None, 50, 40)	0
LSTM	(None, 100)	56,400
Dense	(None, 1)	101
Total parameters: 256,501		
Trainable parameters: 256,501		
Non-trainable parameters: 0		

##### LSTM

It is a popular recurrent neural network. The recurring module is responsible for learning long-term dependencies of text. In this paper, we select the value of optimal hyper-parameters based on experiments. From Fig. 10, we can observe the layered architecture of the LSTM model, while Table 4 represents the hyper-parameter settings for LSTM.

##### Bi-LSTM

Bi-LSTM classifier uses two LSTM classifiers for training the input sequence. Table 6 represents the layered architecture of Bi-LSTM used in the proposed work, and Table 7 represents the experimental value set-up used to achieve the highest performance of the model.

**Table 7:** Hyper-parameter settings of Bi-LSTM

Hyper-parameter	Values
Number of dense layers	1
Dropout rate	0.3
Optimizer	Adam
Activation function	Sigmoid
Loss function	Binary cross-entropy
Number of epochs	10
Batch size	64

### Image classification

#### VGG16

**Fig. 11:**
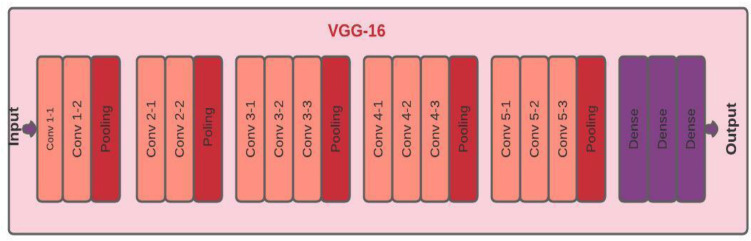
VGG16 architecture [77]

For image classification, we adopt VGG16 model. VGG16 is one of the most preferred CNN architectures in the recent past, having 16 convolution layers. The detailed framework of the VGG 16 model is shown in Fig. 10. These 16 convolution layers are divided into 6 layers. The first convolution layer (layer 1 and 2) has 64 channels of 3*3 kernel with padding one, and after the max-pooling, the size of these layers is 224. The second layer (layer 3 and 4) has 128 channels of 3*3 kernel having size 112. The third layer (layer 5, 6 and 7) has 256 channels of 3*3 kernel having size 56, fourth layer (layer 8, 9 and 10) have 512 channels of 3*3 kernel which size is 28, fifth layer (layer 11, 12 and 13) have 512 channels of 3*3 kernel having size 7 and the last layer is entirely used as a dense layer. Table 8 represents the values of the hyper-parameters used during experiments to achieve maximum accuracy (Fig. 11).

**Table 8:** Hyper-parameter settings for VGG 16 model

Hyper-parameter	VGG16
Number of convolution layers	16
Number of max pooling layers	5
Number of dense layers	2
Optimizer	Adam
Activation function	Softmax
Loss function	Binary cross-entropy
Number of epochs	10
Batch size	32

#### Resnet-50

**Fig. 12:**
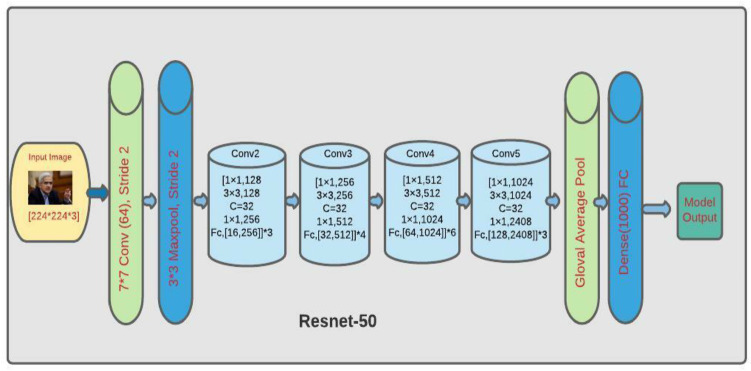
Resnet-50 architecture [78]

The Resnet-50 architecture consists of 50 layers (see Fig. 12). The Resnet-50 model consists 5 stages, each one of which has convolutional layers and identity blocks. Each convolutional block and identity block consist of a three convolutional layer separately. The same structure repeats for 50 layers. This is the reason that Resnet-50 has over million trainable parameters.

## Results analysis

This is the specification of the GPU system:-Intel Xeon Gold 5222 3.8 GHz Processor, Dual Nvidia Quadro RTX4000,8 GB Graphics, Windows 10 Pro Operating System, 128 GB 8 16 GB DDR4 2933 Memory(RAM), 1 TB 7200 RPM SATA Hard Disk.

### Text modality results

**Fig. 13:**
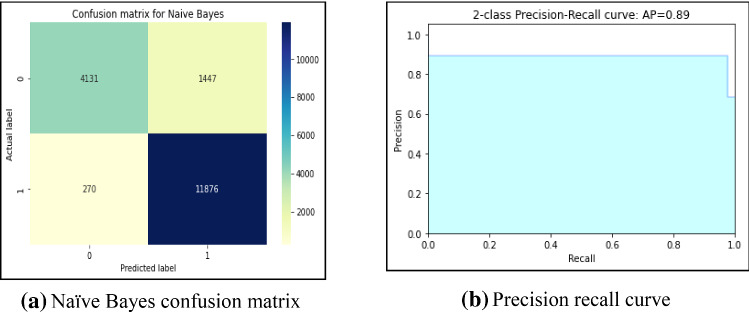
**a** Naïve Bayes confusion matrix. **b** Precision-recall curve

**Fig. 14:**
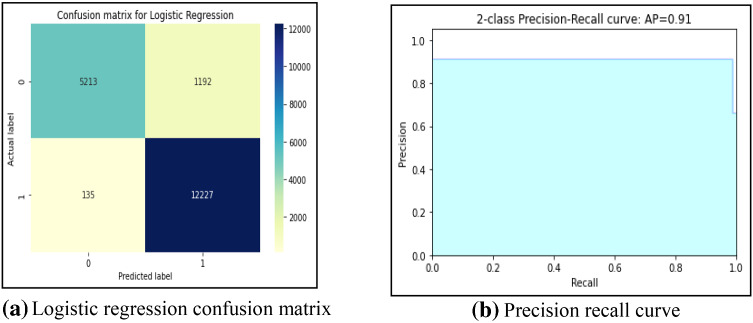
**a** Logistic regression confusion matrix. **b** Precision-recall curve

**Fig. 15:**
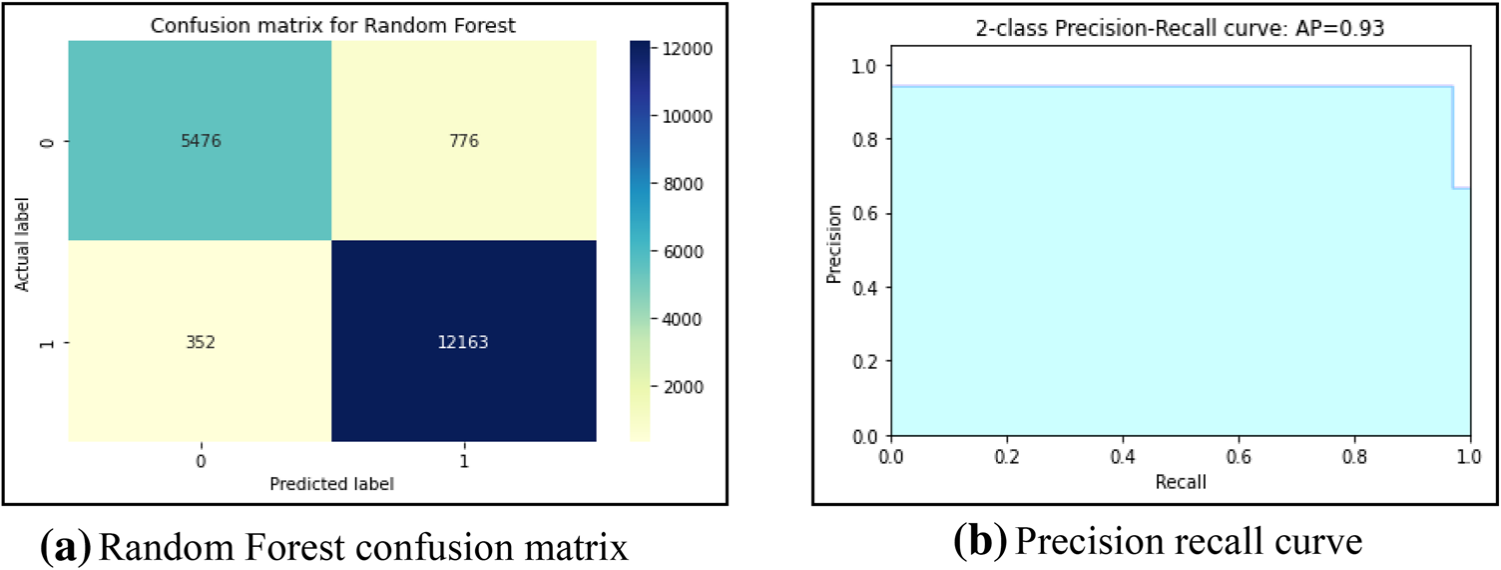
**a** Random Forest confusion matrix. **b** Precision-recall curve

**Fig. 16:**
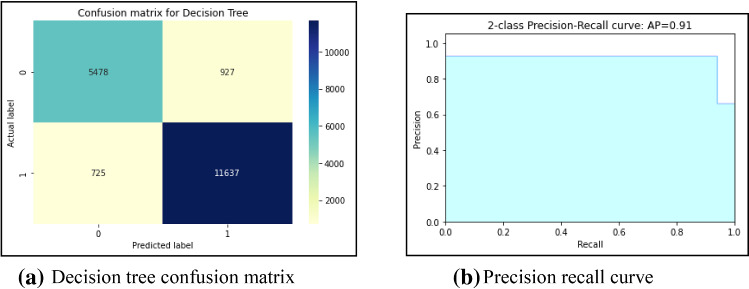
**a** Decision Tree confusion matrix. **b** Precision-recall curve

**Fig. 17:**
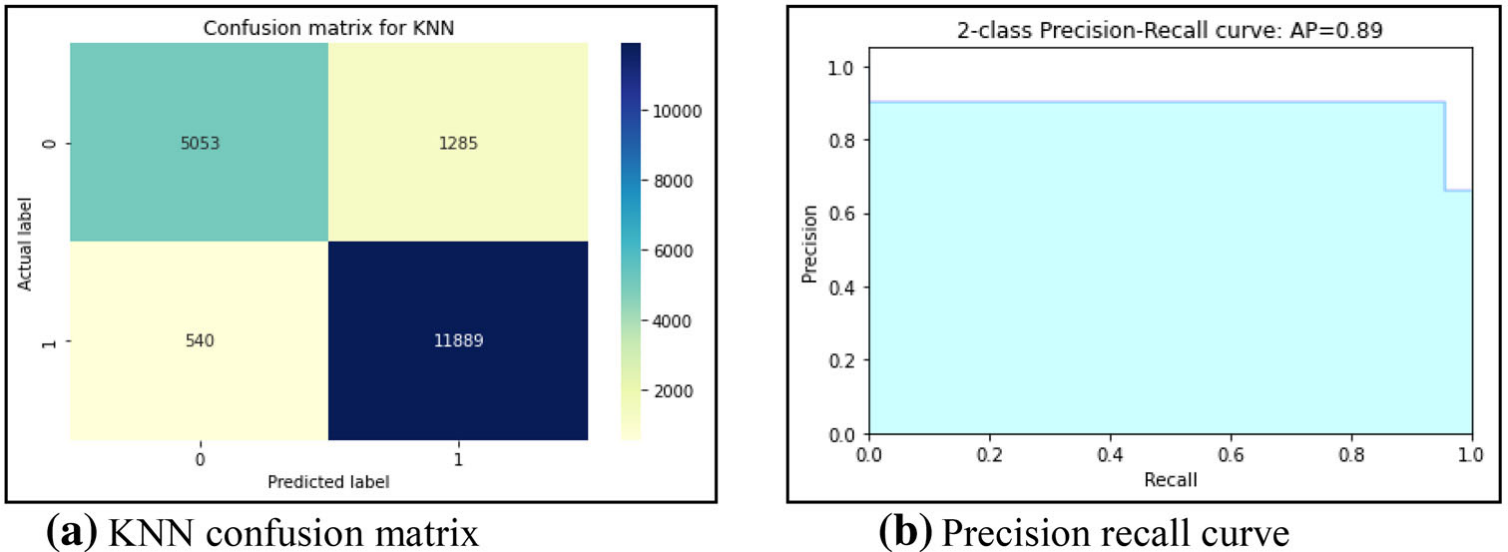
**a** KNN confusion matrix. **b** Precision-recall curve

**Fig. 18:**
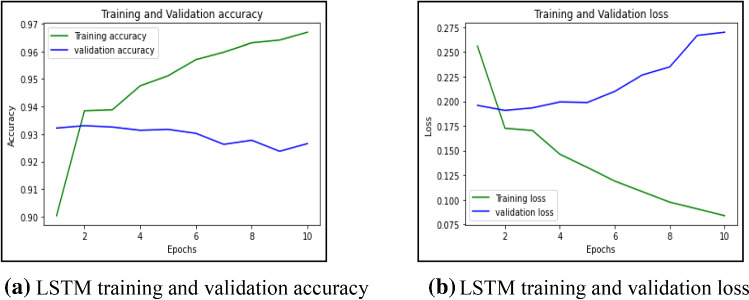
**a** LSTM training and validation accuracy. **b** LSTM training and validation loss

**Fig. 19:**
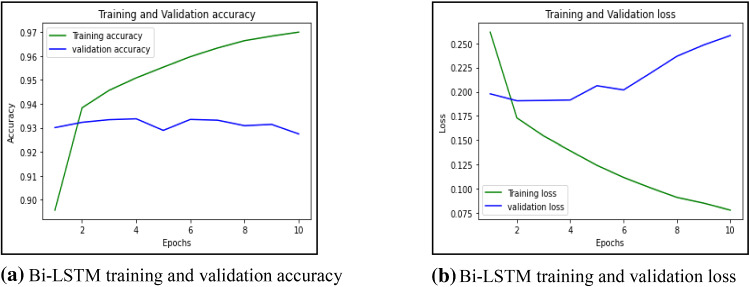
**a** Bi-LSTM training and validation accuracy. **b** Bi-LSTM training and validation loss

First, this work had implemented several ML models to compute the performance. Tenfold cross-validation is applied to analyze the performance. Multinomial Naive Bayes (MNB), logistic regression (LR), K-nearest neighbour (KNN), Random Forest (RF), Decision Tree (DT) are implemented on the IFND dataset. Multinomial Naïve Bayes classifier achieved an accuracy of 87.5%. The confusion matrix of the MNB classifier is represented in Fig. 13a. The value of Confusion matrices of other ML models is shown in Figs. 14a, 15a, 16a, and 17a. Figure 15a represents that random forest classifiers predict more accurate results in comparison to other ML models. True-positive value and true-negative value of random forest classifier are high means if the news is false, it shows false, and in case of true news, it predicts the same. Precision-recall graph is also important to analyze the performance of the dataset. Average precision (AP) is generally the weighted average precision across all thresholds. Precision-recall graph works well for binary classification problems where the dataset is imbalanced. Random Forest classifier (Fig. 15b) achieved an average precision value of 0.93, which is greater in comparison to another classifier which is shown in Figs. 13b, 14b, 16b, and 17b. The higher the value of the precision-recall curve indicates, the better classifier performance for a given task. In this paper, we also implemented various deep-learning models. A deep-learning model can learn features automatically. LSTM classifier achieved 92.6% accuracy, and Bi-LSTM achieved 92.7% accuracy. Figure 18a, b represents the accuracy and loss of the LSTM model, while Fig. 19a, b represents the accuracy and loss of the Bi-LSTM model. The Pypolt module of the Matplotlib library has been used to represent the learning curve of the LSTM and Bi-LSTM classifier. Figure 18a represents the training and validation accuracy of the LSTM classifier. The training accuracy increases with the epochs while the validation accuracy remains almost the same. Similarly, Fig. 18b represents training loss values decrease with an increase in epochs’ values, which indicates that the model learns to classify the articles better, but validation loss increases with an increase in epochs’ values. Figure 19a represents that training accuracy improves with the increased value of epochs while validation accuracy remains almost constant. Figure 19b represents the smooth decrease in training loss while validation loss increases with epochs.

**Fig. 20:**
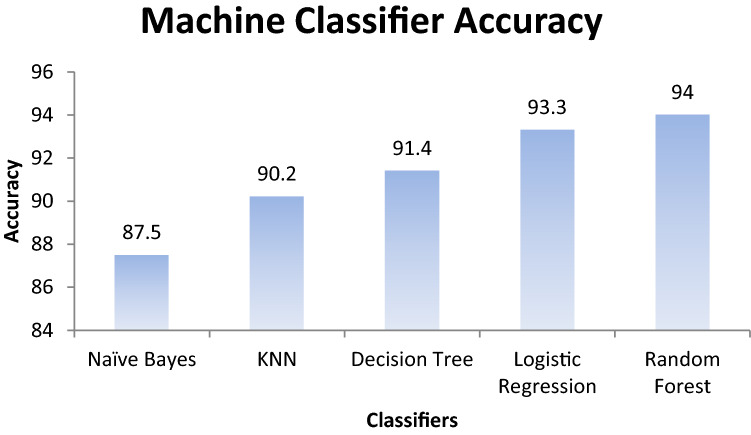
Machine learning classifier accuracy

Figure 20 shows the comparison of the existing machine learning classifier. Results indicate that the Random forest classifier achieved the highest of 94% accuracy in text classification. Deep-learning model LSTM achieved 92.6% accuracy, and the Bi-LSTM classifier obtained 92.7% accuracy.

## Comparison with existing dataset (textual features)

To show the effectiveness of the IFND dataset, the Mediaeval and LIAR dataset is used. The LIAR dataset consists of a total training dataset of size 11,554, and a testing dataset size of 1760. Only the statement and label of dataset are used for computation. Dataset is converted from six-label to two-label for processing. Tf-idf embedding is applied for feature extraction than different machine learning classifier is applied. In the case of a deep-learning classifier, one-hot encoding is applied, then LSTM and BI-LSTM are implemented.

In the case of the Medieval-2016 dataset, the text consists of many languages, so, first, Google translates library is used to convert text into the English language. After that, there were specific tweets that are not translated properly, so we removed those tweets. So a total of 10,914 tweet texts were used for training and 1760 for testing. Only tweet text and label are considered for implementation. The same machine-learning and deep-learning model architecture used for IFND is used in Mediaeval also. Tables 9 and 10 represent the comparison of these datasets. The proposed IFND dataset performed better due to the large training and testing size.

**Table 9:** Accuracy comparison of three different dataset using machine-learning models

Models	Accuracy		
	Medieval	LIAR	IFND
Naïve Bayes classifier	71.0	70.2	87.5
K-nearest neighbor	64.7	46.1	90.2
Decision Tree	68.3	62.1	91.4
Logistic regression	72.2	70.3	93.3
Random Forest	70.8	68.6	94.0

**Table 10:** Accuracy comparison of three different dataset using deep-learning models

Models	Accuracy		
	Medieval	LIAR	IFND
LSTM	44.5	58.6	92.6
Bi-LSTM	44.8	48.5	92.7

### Image modality results

**Fig. 21:**
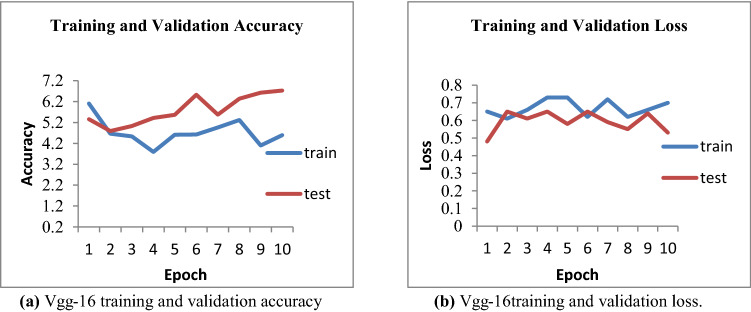
**a** Vgg-16 training and validation accuracy. **b** Vgg-16 training and validation loss

For VGG 16 model, we used the sigmoid activation function. Adam optimizer is used. All images were resized to 232 × 232 size. Figure 21a represents that the curve of training and the testing accuracy is not smooth. They are fluctuating concerning epochs values. The vgg-16 model achieved 65.3% testing accuracy. The loss of the VGG-16 model is shown in Fig. 21b. The validation loss is minimum at epoch 2 with a value of 4.79 and maximum at epoch 10 with a value of 6.73. Training loss fluctuates from a minimum of 3.79 to a maximum of 6.11. In the case of Resnet-50, the IFND model achieved 76.6% accuracy when the image size is 256*256. Table 11 represents the comparison of the IFND dataset with the existing CASIA dataset and Mediaeval-2016 dataset. CASIA dataset consists of a total of 515 images, while the Mediaeval-2016 dataset consists of a total of 10,992 images. Only images are used for comparison. The proposed IFND dataset works well in the case of both 256*256 image size as input and 32*32 image size.

**Table 11:** Accuracy comparison of images using in three different dataset

Models	Accuracy			
	Mediaeval	CASIA	IFND (256*256)	IFND (32*32)
VGG-16	46.1	58.9	65.3	50.0
Resnet-50	53.6	53	76.6	70.8

### Multi-modal results

**Fig. 22:**
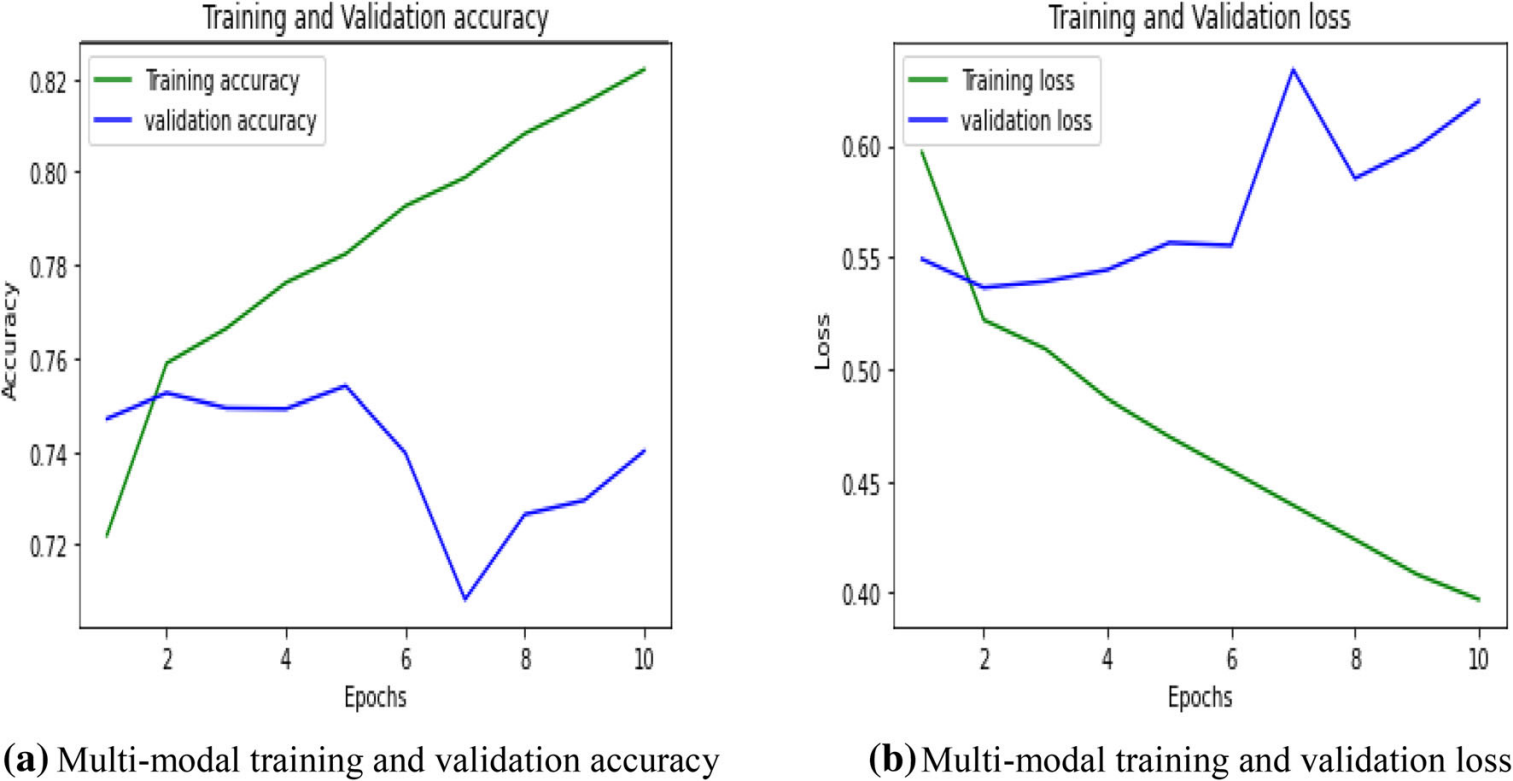
**a** Multi-modal training and validation accuracy. **b** Multi-modal training and validation loss

In this paper, text and visual features are fused together to determine the outcome of the proposed model. The LSTM model is trained for text analysis, and for image analysis, VGG 16 is used. The features obtained through both the channels are merged to calculate the accuracy of the proposed multi-modal approach. IFND dataset achieved 74% accuracy in the case of 256*256 image size. Accuracy is dropped to 66% when image size is reduced to 32*32. So, we concluded that better prediction depends on image size also. Table 12 shows the architecture used for training multi-modal for fake news detection. Figure 22a depicts that training accuracy increases with an increase in the value of epochs while validation accuracy increases with an initial value of epochs, but at epoch7, it reduces drastically. After seven epochs, it again shows an increasing trend. Figure 22b represents that training loss decreases with an increase in value of epoch, but validation loss increases suddenly at epoch7 due to a decrease in validation accuracy. After that, it reduces and does not fluctuate much. Table 13 shows the comparison of the existing dataset with the IFND dataset using a multi-modal approach. Results show the effectiveness of our dataset. When the image size is 32*32, the accuracy of the IFND dataset is reduced. It is due to a decrease in the value of false-negative and an increase in false-positive values. By reducing the resolution of the image, the high-frequency information is lost, which resulted in a decrease in the value of accuracy.

**Table 12:** Layered architecture of multi-modal approach

Layer (type)	Output shape	Param #	Connected to
input_4 (InputLayer)	[(None, 50)]	0	
embedding (Embedding)	(None, 50, 40)	200,000	input_4[0][0]
dropout (Dropout)	(None, 50, 40)	0	embedding[0][0]
input_3 (InputLayer)	[(None, 4096)]	0	
lstm (LSTM)	(None, 100)	56,400	dropout[0][0]
dense (Dense)	(None, 32)	131,104	input_3[0][0]
dense_1 (Dense)	(None, 32)	3232	lstm[0][0]
add (Add)	(None, 32)	0	dense[0][0] dense_1[0][0]
flatten (Flatten)	(None, 32)	0	add[0][0]
dense_2 (Dense)	(None, 1)	33	flatten[0][0]
Total params: 390,769			
Trainable params: 390,769			
Non-trainable params: 0

**Table 13:** Comparison of multi-modal approach

Dataset	Classifier	Accuracy
Mediaeval 2016	LSTM + VGG16	0.70
IFND (224*224)	LSTM + VGG16	0.74
IFND (32*32)	LSTM + VGG16	0.66

## Applications

There are various fields where this IFND dataset can be used.

- *Indian government* This dataset is specially designed for the Indian subcontinent. The Indian government could use this dataset to keep the record of fake news and also help in determining the trend of fake news. Fake news is generally generated in large amounts during election time to generate biased opinions.
- *Media* Media journalists could use this dataset to check the authenticity of the news. They can check whether a viral image with the claim is already published with different content or not.
- *General user *User can also use this dataset to check the veracity of the claim because this dataset collects the news from reliable sources.
- *Researchers* Researchers could use this IFND dataset for further fake news detection work.

## Conclusion

In this study, a benchmark dataset from an Indian perspective for fake news detection is introduced. Based on existing research, this is the first Indian large-scale dataset that consists of news from the year 2013 to 2021. This dataset contains image content for every news headline. This dataset will help other researchers to perform experiments and understand the Indian context better. LDA topic modelling approach is employed to determine the category of news statements. All news are manually verified to extract news only related to India. Three manual annotators have done this verification. The augmentation technique is applied to increase the size of fake datasets, which helps in increasing the performance of the model.

The limitation of the proposed approach is that VGG-16 and Resnet-50 classifiers take more time during training in comparison to the machine learning approach. There is also a need to collect data from social networking websites. Social-contextual information is missing, which will help to keep track of the account of the person who spreads fake news. As next, there are two areas where this research will be extended. First, the dataset can be extended with more context information like author’s information, website credibility and social context. Second, audio and video information could be added in the dataset. For other researchers, this comprehensive dataset will be a valuable asset for more research on fake news detection.
